# How to Measure for a Truss

**Published:** 1900-07-28

**Authors:** 


### HOW TO MEASURE FOR A TRUSS.

In many cases it is desirable for a surgeon to fit his patient with a truss instead of handing the case over to the instrument maker, as is so commonly done. Mr. McAdam Eccles[1] gives directions for the measurement of a patient for a truss. The patient should always be lying down. The surgeon should stand on the patient's right, and, taking a flexible measure, he slips the tape underneath the patient's body from the patient's left, allowing the figures on the tape to lie next to the patient's skin, the lower numbers to the patient's right and the higher on the patient's left. Placing his left thumb at the commencement of the numbers he brings the tape round so that it lies over the site of the deep abdominal ring on the patient's right side. The other part of the tape he takes in his right hand, with his right thumb against the figures, and brings the two thumbs together, drawing the tape fairly tightly. Then, dropping the measure where it is held by the left hand, and maintaining the hold on the tape with his right thumb, he pulls the tape away from the patient and reads off the number at the position of his thumb. The length thus obtained is that required for an ordinary single truss. If a double one is wanted it is well to add an inch to the measurement, because a double truss does not stretch at all, whereas the strap of a single one stretches considerably. It is important to remember that the line along which the tape should lie should run across the base of the sacrum behind, half way between the crest of the ilium and the top of the great trochanter at the sides, and at a higher level than the symphisis pubes in front. The truss maker will require to know, in addition to the measurement obtained as above, the variety of truss (inguinal, femoral, or umbilical), and if it be a single truss the side for which it is required.

[1] Clinical Jour., July 11.

